# Wildlife–Vehicle Collisions and Mitigation: Current Status and Factor Analysis in South Korea

**DOI:** 10.3390/ani14203012

**Published:** 2024-10-18

**Authors:** Ju-Won Hwang, Yeong-Seok Jo

**Affiliations:** Department of Biology Education, Daegu University, Gyeongsan 38453, Republic of Korea; oklhy1230@naver.com

**Keywords:** mitigation strategies, road-kill, road features, species composition

## Abstract

**Simple Summary:**

Habitat loss and fragmentation from extensive road development have escalated wildlife–vehicle collisions (WVCs) as a major cause of wildlife mortality. This study covers 9 years of WVC data across South Korea, examining species involved, seasonal and regional variations, and road factors affecting WVCs, to analyze impacts and propose mitigation strategies. Over 4561 km of roads were monitored, yielding insights into the connection between WVCs, road characteristics, and species-specific patterns. The study identified 13,606 WVCs involving 143 terrestrial vertebrate species, with detailed patterns and models for the top seven mammal species. Spatial differences in WVCs appeared more related to wildlife habitats, but some road features correlated variably with WVC frequency, highlighting complex prevention challenges. Effective mitigation requires comprehensive strategies tailored to species traits, seasonality, and road types.

**Abstract:**

Severe habitat loss and fragmentation due to extensive road development have escalated wildlife–vehicle collisions (WVCs) as one of the major causes of wildlife mortality. This study, spanning 9 years from 2009 to 2017, presents comprehensive WVC data in South Korea, including species composition, seasonal and regional patterns, and road factors influencing WVCs, aiming to analyze their impact and propose effective mitigation strategies. We collected WVC data with road variables for 9 years from 4561 km of nationwide monitoring road sections and analyzed the data to understand the relationship between WVCs and road characteristics, as well as species-specific patterns. A nationwide survey identified 13,606 WVCs involving 143 terrestrial vertebrate species, and patterns and models of the top seven mammal species (raccoon dog (*Nyctereutes procyonoides*), Siberian chipmunk (*Eutamias sibiricus*), Siberian weasel (*Mustela sibirica*), water deer (*Hydropotes inermis*), red squirrel (*Sciurus vulgaris*), Korean hare (*Lepus coreanus*), and leopard cat (*Prionailurus bengalensis*)) were presented. Patterns revealed declines in WVCs overall, except for water deer. Although spatial differences in WVCs seemed linked more to wildlife habitats, certain road features correlated both positively or negatively with WVC frequency, highlighting complexities in the effectiveness of preventative measures. For effective mitigation and prevention of WVCs, comprehensive strategies considering species traits, seasonality, and road types should be implemented

## 1. Introduction

Natural ecological corridors for animal migrations across the entire range contribute to maintaining their populations in these territories [1,2]. This increases the potential risks of animal death due to human activity, which deserves close attention. Since wildlife–vehicle collision (WVC) was first recognized as a new cause of wildlife mortality [3], WVCs have drastically increased worldwide. While road systems are necessary infrastructure for human life, they can often act as ecological traps for wildlife populations [4,5]. In addition to habitat fragmentation caused by road systems, the roads themself lead to WVCs as wildlife moves through fragmented habitats [5,6,7,8,9]. Despite the drastic increase in traffic volume and road networks resulting in WVCs, studies on WVCs have been insufficient or inadequate [10]. Although the investigation of WVCs with road features is necessary for reducing or preventing WVCs, the current situation of WVCs has been rarely surveyed in some countries [11], and the road factors affecting WVCs have been little studied.

In South Korea, rapid economic growth after the Korean War (1950–1953) led to severe habitat decline and fragmentation for wildlife due to massive infrastructure development [12]. As of 2020, the total road area in South Korea covers 1712 km^2^, accounting for approximately 1.7% of the country’s total terrestrial area [13]. Paved roads have severely expanded not only within major cities but also into remote mountainous areas including national parks and preserved areas [14]. Due to continuous increases in the number of vehicles and traffic volume, WVCs have become one of the crucial wildlife mortality causes in South Korea [15].

While there have been some studies on the seasonal tendency of WVCs in South Korea [16,17] and WVCs in specific regions [9,16,18,19,20,21,22,23,24,25], a nationwide survey of WVCs has been rarely conducted in South Korea [26]. Limited studies on specific regions might introduce biases regarding species composition, seasonality, or contributing factors influencing WVCs [27].

Here, we present 9 years of WVC data from 2009 to 2017. We aim to report the results of nationwide WVC occurrences in South Korea. We hypothesize that the differences in WVCs among species, seasons, and regions, as well as the combined effects of road features for each species, are significant. Through this nationwide WVC dataset, we attempt to identify the wildlife species involved in collisions, the seasonal and regional frequency of WVCs, and the road factors influencing WVC occurrence. Therefore, this study aims to quantitatively investigate whether road variables influence WVCs and whether variations occur depending on the species. In the context of tendency and analysis of WVCs in South Korea, our goal is to identify the causes of WVCs and propose efficient mitigation strategies against WVCs.

## 2. Materials and Methods

### 2.1. Study Area

**Figure 1:**
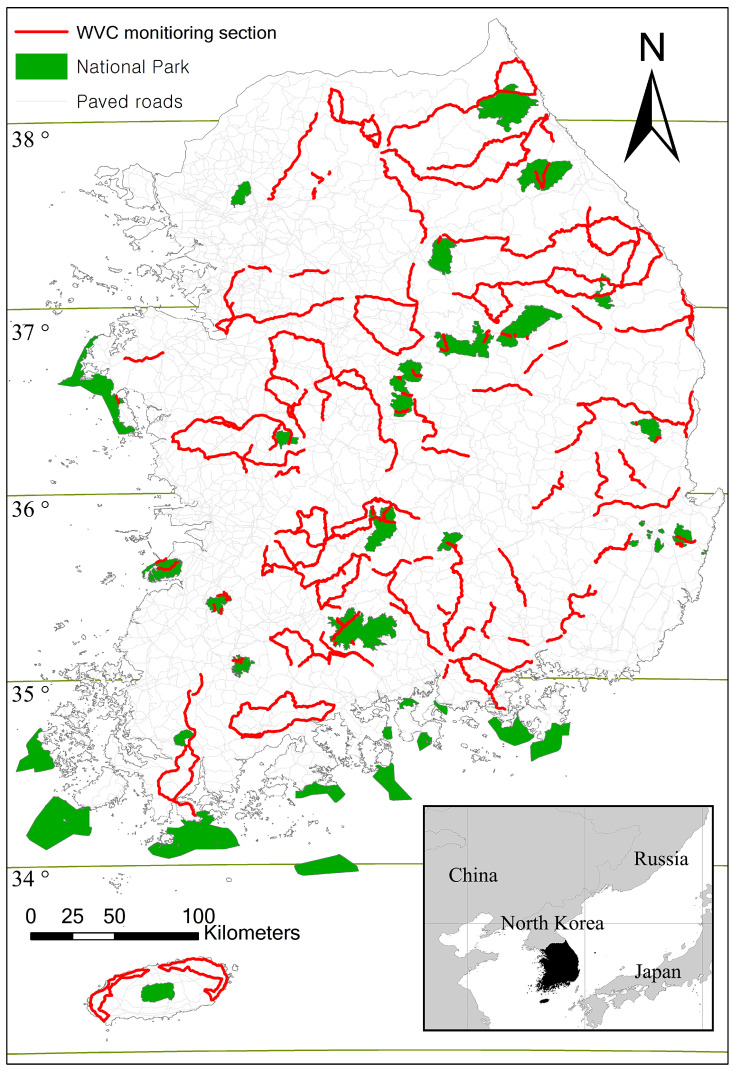
Location of wildlife–vehicle collision (WVC) monitoring road sections on major roads (expressways, national, provincial, and county roads) in South Korea.

In 2007, as part of the Wildlife Survey, the National Institute of Biological Resources (NIBR) conducted a three-month pilot study on a nationwide WVC survey [28]. In 2008, NIBR and seven regional environmental offices in South Korea designated national WVC monitoring roads [26]. Additionally, the Korean National Park Service (KNPS) and Korea Expressway Corporation designated WVC monitoring sectors along roads in national parks and highways, respectively [26]. Each monitoring sector was selected by well-trained wildlife biologists in each regional environmental office based on field conditions such as approachability and safety [27]. The WVC monitoring road sectors encompassed a total of 4561 km covering all 9 provinces in South Korea (Figure 1). Local environmental offices selected the monitoring road sections in consultation with NIBR. Approximately 4% of paved roads, including highways, national roads, provincial roads, and city or county roads, were sampled in each province. The regional survey roads covered Gyeonggi Province including Seoul Special City (338 km, 7.41%), Gangwon Province (1074 km, 23.55%), Chungcheongbuk Province (359 km, 7.87%), Chungcheongnam Province including Daejeon Metropolitan City (178 km, 3.9%), Gyeongsangbuk Province including Daegu Metropolitan City (560 km, 12.28%), Gyeongsangnam Province including Ulsan and Busan Metropolitan Cities (493 km, 10.81%), Jeollabuk Province (534 km, 11.71%), Jeollanam Province including Gwangju Metropolitan City (485 km, 10.63%), and Jeju Island (222 km, 4.87%). The WVC survey road sections in national parks and highways covered 291 km (6.38%) and 27 km (0.59%), respectively. Generally, the speed limits for two-lane roads are 60 km/h, and for four-lane roads, they are 80 km/h.

### 2.2. WVC Data Collection

From 2009 to 2017, we collected GPS coordinates of WVCs along the monitored roads. The field surveys were conducted monthly by 27 well-trained wildlife biologists through driving in a vehicle to visually inspect and identify the carcasses of road-killed animals. At least two wildlife biologists (a driver and a spotter) investigated WVCs from 9 a.m. to 5 p.m. They drove monitored segments through planned circuits, ensuring all monitored segments were surveyed during the same period of the month. All surveyors observed both the minimum and maximum speed limits. Given the various road conditions such as differences in the number of lanes, speed limits, fences, and road shoulders, surveyors needed to improvise to ensure their safety while collecting data. Speed regulations vary by administrative region, so both minimal and maximum speeds on the surveyed roads also differed. When a road-killed animal could not be identified readily in the field, detailed photographs were taken and further identification was conducted by the mammal collection manager at NIBR. All checked carcasses were removed from the roads to prevent duplication.

Because the survey was not designed for further analysis but for a simple report and statistics, the collected field data only included date, GPS coordinates, and species. Therefore, we obtained the additional data through the road-view service of Naver Maps (map.naver.com) or Kakao Maps (map.kakao.com). Using the road view of the exact times and locations of WVCs, we collected additional road variables such as road width, shoulder, wildlife fences (guardrails), median strips (central reservation or fence), and speed limits. Spatial data (locations of WVC occurrences) were categorized based on administrative regions and latitudes. The road width was recorded as the number of lanes; the presence of a road shoulder was considered when it provided sufficient width for one car size (about 3 m); the presence or absence of a median strip was recorded; the number of wildlife fences were recorded as ‘0’ when not present, ‘1’ when present on one side, and ‘2’ when present on both sides; the speed limits at the location of WVCs were found through road speed signs. When nearby speed signs were not identified, we recorded a speed limit based on Article 19 of the Enforcement Regulations of the Road Traffic Act (60 km/h for two-lane roads and 80 km/h for four-lane roads).

To identify the relation between WVCs and traffic intensity, we used daily traffic data from 3709 traffic monitoring points (www.road.re.kr accessed on 21 March 2024). Using the ‘spatial join’ function in ArcGIS ver. 1.6 (ESRI Inc., 2018), we appended daily traffic volume to WVC points. Additionally, altitudes of WVCs were included as a road variable. We utilized ecological and natural maps (nie-ecobank.kr accessed on 21 March 2024) to recognize the impact of vegetation. Both vegetation rank (rank 1 is well-preserved vegetation, rank 2 is intermediate, and rank 3 is rather disturbed vegetation) and the distance from the vegetation to WVCs were added to WVC points using the ‘spatial join’ function with the ‘near’ function in ArcGIS. To identify urbanization effects on WVCs, we applied a 1 km resolution human footprint index (sedac.ciesin.columbia.edu accessed on 21 March 2024), which covers human population pressure, anthropogenic land use, and human access. Outlier GPS coordinates that significantly deviated from the actual roads were excluded. In addition, domestic animals such as dogs and cats were not included in the analysis.

### 2.3. WVC Data Analysis

We used R 4.2.1 (R Core Team, 2021) software to generate a generalized linear model (GLM), which examines the relationship between the number of WVCs and road variables (median strips, wildlife fences, presence of road shoulders, road width, traffic volume, altitude, distance from vegetation, and vegetation rank). The location of WVCs was treated as a random effect. The WVC points were categorized by road variables, and the number of WVC cases was regarded as a dependent variable. To identify differences among species, multiple factor analysis (MFA) was applied using the R package “FactoMineR” [29]. For each species model, we used the R package “LME4” [30] to generate linear mixed-effects models with road variables to explain the locations where WVCs of specific species occurred (presence) compared to the locations where WVCs of other species occurred (pseudo-absence). The pseudo-absence points were plausible WVC points, but target species was not identified. To avoid overlap of multiple species in ‘presence’ points, we excluded WVCs within a 1 km radius of presence points (1 km is based on average radius of home range for wild boar, Sus scrofa in South Korea) [12]. We compared the generated models using the conditional AIC (cAIC, adjusted AIC for mixed-effects models) in the R package “cAIC4” [31]. Only models with high support (ΔAIC < 2) were presented for further interpretation [32]. The model average was calculated with R package “MuMIn” [33].

## 3. Results

Over the 9 years from 2009 to 2017, a nationwide survey of wildlife WVCs identified a total of 13,606 cases, involving 143 species of terrestrial vertebrates (Table S1). The top 10 terrestrial vertebrate species with the highest detection frequency were as follows: raccoon dog *Nyctereutes procyonoides* (Gray, 1834) at 11.34%, Siberian chipmunk *Eutamias sibiricus* (Laxmann, 1769) at 11.30%, Siberian weasel *Mustela sibirica* Pallas, 1773 at 9.36%, water deer *Hydropotes inermis* Swinhoe, 1870 at 9.33%, red squirrel *Sciurus vulgaris* Linnaeus, 1758 at 5.24%, oriental fire-bellied toad *Bombina orientalis* (Boulenger, 1890) at 4.99%, pheasant *Phasianus colchicus* Linnaeus, 1758 at 4.09%, tiger keelback *Rhabdophis tigrinus* (H. Boie, 1826) at 3.24%, Dybowski’s frog *Rana dybowskii* Günther, 1876 at 2.96%, and oriental turtle-dove *Streptopelia orientalis* (Latham, 1790) at 2.53%. Mammal species with WVC frequencies over 1% included 7 species: raccoon dog, Siberian chipmunk, Siberian weasel, water deer, red squirrel, Korean hare (*Lepus coreanus* Thomas, 1892, 1.63%), and leopard cat (*Prionailurus bengalensis* (Kerr, 1792), 1.02%).

**Figure 2:**
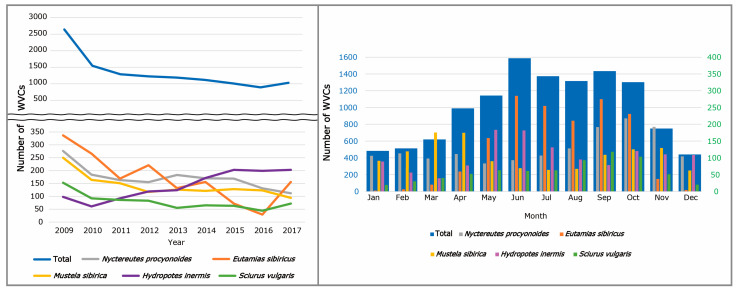
The annual number of wildlife–vehicle collisions (WVCs) involving the top five mammal species (**left**), and monthly WVCs involving top five mammal species (**right**) from 2009 to 2017 in South Korea. The blue numbers indicate total WVCs and the green numbers indicate WVCs of each species.

Following a drastic reduction in WVCs in 2010, the number of WVCs exhibited a consistent decline (Figure 2). Despite this decline, WVCs involving water deer exhibited an approximate two-fold increase after 2010. In contrast to water deer, WVC numbers for raccoon dogs, Siberian chipmunks, Siberian weasels, and red squirrels represented a decrease (Figure 2). Except for these 5 species, there were insignificant changes observed for the remaining 138 species.

**Figure 3:**
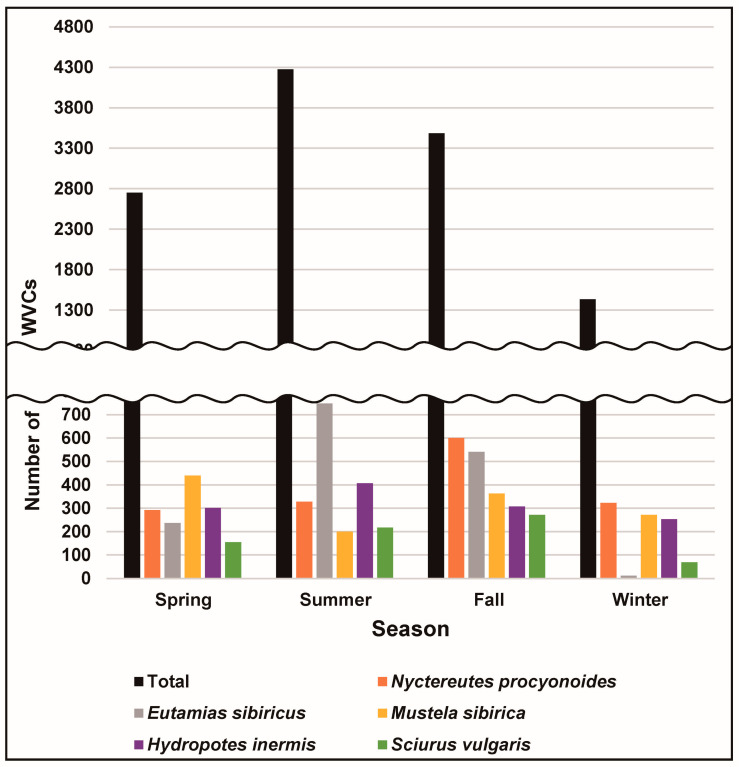
The seasonal difference in wildlife–vehicle collisions (WVCs) involving top five mammal species from 2009 to 2017 in South Korea. Spring is March–May, summer is June–August, fall is September–November, and winter is December–February.

While monthly WVCs exhibited a peak occurrence between June and October, constituting 58.6% of the total number of WVCs, a clear decline from November to February followed, accounting for 18.2% of the total (Figure 2). Seasonally, WVCs were most prevalent from summer through autumn, followed by a gradual decline during autumn and winter, with a subsequent increase in spring (Figure 3).

**Figure 4:**
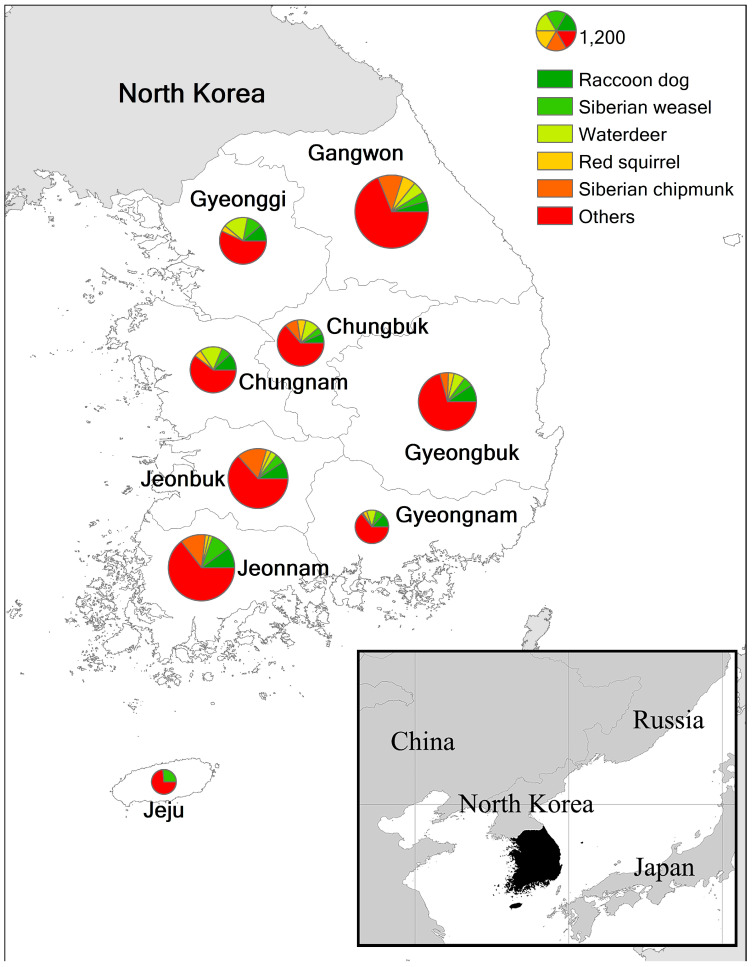
Provincial map with occurrence of wildlife–vehicle collisions (WVCs) involving top five mammal species from 2009 to 2017 in South Korea. The size of pie chart represents the number of WVCs.

**Figure 5:**
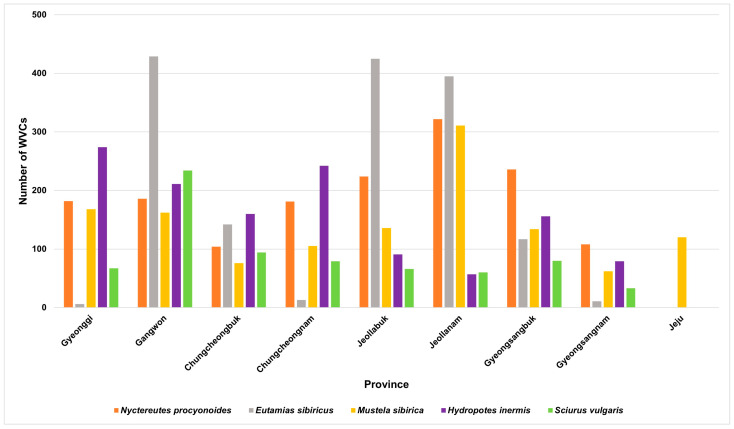
Provincial difference in wildlife–vehicle collisions (WVCs) involving top five mammal species from 2009 to 2017 in South Korea.

Among the nine provinces, Gangwon Province exhibited the highest frequency of WVCs, while Jeju Island had the lowest (Figure 4 and Figure 5). South Korea latitudes range between 38° and 33° north, and the number of WVCs decreased toward the northern and southern extremities of the country. On Jeju Island, unlike the mainland of Korea, Siberian weasel was a major victim of WVCs (Table 1).

Wildlife fences, road shoulders, road width, altitude, vegetation rank, and urbanization were consistently included in the top six linear mixed-effects models. Speed limit was confirmed in four out of the six models. The presence of median strip was observed in two models. The traffic volume and distance from vegetation were each identified in only one model, while the month variable was excluded from all selected models (Table 2). Based on MFA, WVC patterns for seven mammal species were distinguished (Figure S1). The specific statuses and linear models for the top seven mammal species involved in WVCs in South Korea are as follows.

### 3.1. WVCs Involving Raccoon Dogs

**Figure 6:**
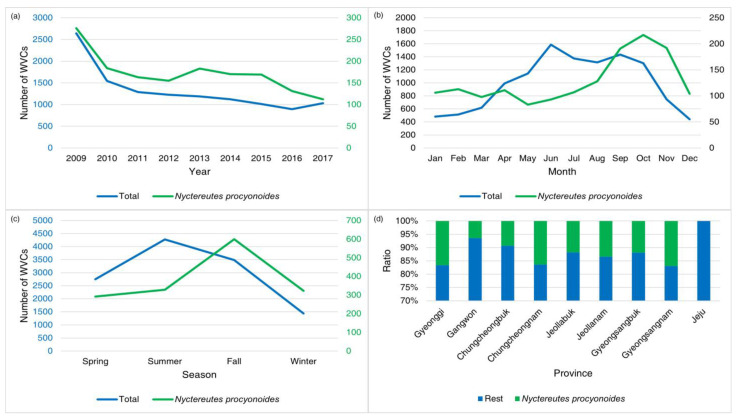
The annual (**a**), monthly (**b**), seasonal (**c**), and provincial (**d**) patterns of wildlife–vehicle collisions (WVCs) involving raccoon dogs (*Nyctereutes procyonoides*) from 2009 to 2017 in South Korea. The blue numbers indicate total WVCs and the green numbers indicate WVCs of raccoon dogs.

From 2008 to 2017, the raccoon dog was the most frequent species in WVCs (11.34 % of total WVCs). Apart from an increase in 2013, the number of WVCs involving raccoon dogs displayed a declining trend (Figure 6). The peak occurrence of raccoon dog WVCs was concentrated between September and November, particularly high during autumn (Figure 6). During spring, summer, and winter, raccoon dog WVCs remained relatively lower (Figure 6). Except on Jeju Island, WVCs involving raccoon dogs contributed to 7–20% of the total WVCs in the mainland areas. Among these regions, Gyeongsangnam Province reported the highest number of WVCs involving raccoon dogs, while Gangwon Province had the lowest (Figure 5 and Figure 6). Latitudinally, WVCs involving raccoon dogs were prevalent at latitudes 35° and 36° north.

The speed limit, altitude, distance from vegetation, and month were consistently shown in all the top 11 selected models. Urbanization was included in nine models, and the presence of median strip was included in four models. Wildlife fence and traffic volume were confirmed in two models, and road shoulders and road width each appeared in only one model. The number of wildlife fences, the presence of road shoulders, and traffic volume were negatively associated with the number of raccoon dog WVCs, while road width and the presence of a median strip were positively related (Table 3). Additionally, urbanization (higher human footprint index) with lower altitude was associated with a higher number of raccoon dog WVCs.

### 3.2. WVCs Involving Siberian Chipmunks

**Figure 7:**
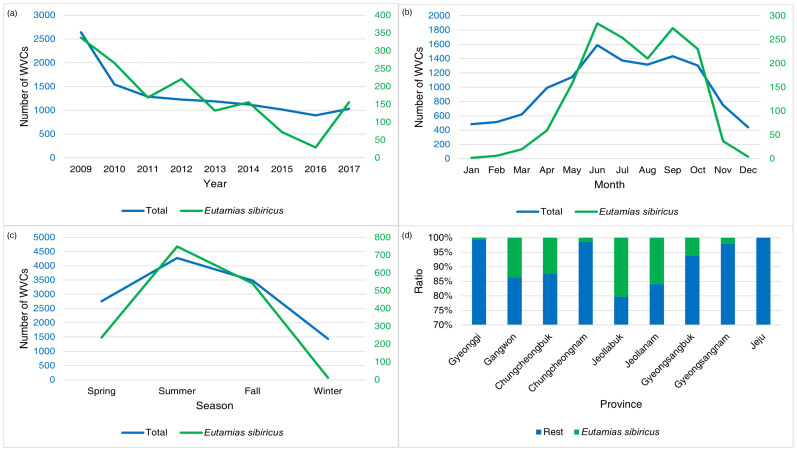
The annual (**a**), monthly (**b**), seasonal (**c**), and provincial (**d**) patterns of wildlife–vehicle collisions (WVCs) involving Siberian chipmunks (Eutamias sibiricus) from 2009 to 2017 in South Korea. The blue numbers indicate total WVCs and the green numbers indicate WVCs of Siberian chipmunks.

Siberian chipmunks were involved in 11.0% of total WVCs, representing the second most frequently detected road-killed animal during the study and accounting for only 0.34% fewer WVCs than raccoon dogs. Annual WVCs involving Siberian chipmunks displayed a declining trend until 2016, but in 2017, there was a substantial five-fold increase (Figure 7). The number of chipmunk WVCs was most prevalent between June and October, with a steep decline in November, followed by a torpor season (December to February) with minimal or no occurrences (Figure 2 and Figure 7). The number of chipmunk WVCs increased during spring, peaked in summer, gradually decreased in autumn, and rarely occurred in winter (Figure 3 and Figure 7). Except on Jeju Island, WVCs involving chipmunks constituted 0.06–25% of the total cases across mainland regions. Jeollabuk Province reported the highest number of WVCs involving chipmunks, while Gyeonggi Province had the lowest (Figure 7). Regions located at latitude 35° north had higher numbers of WVCs involving chipmunks (by at least two-fold) than other regions.

For chipmunk WVCs, 13 models were selected, with road shoulder, speed limit, altitude, and month included in all models (Table 4). Vegetation rank, wildlife fences, and distance from vegetation appeared in 11, 10, and 9 models, respectively. The median strip appeared in three models, road shoulders in five models, and traffic volume in two models. While the median strip was included in three models, both traffic volume and urbanization were identified in only one out of the top 13 models.

### 3.3. WVCs Involving Siberian Weasels

**Figure 8:**
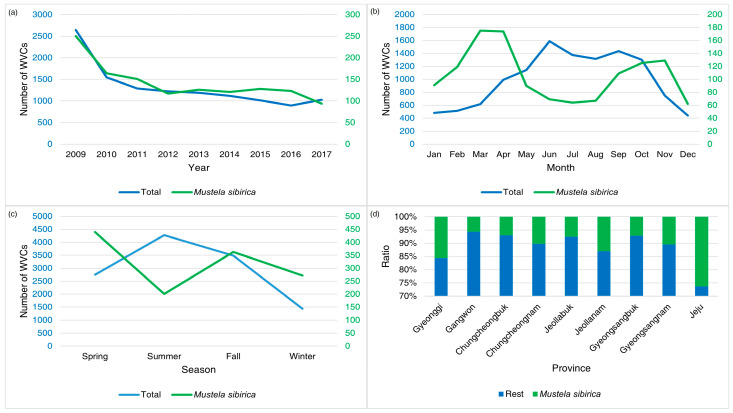
The annual (**a**), monthly (**b**), seasonal (**c**), and provincial (**d**) patterns of wildlife–vehicle collisions (WVCs) involving Siberian weasels (Mustela sibirica) from 2009 to 2017 in South Korea. The blue numbers indicate total WVCs and the green numbers indicate WVCs of Siberian weasels.

The WVCs involving Siberian weasels showed a significant drop in 2010, and the number of WVCs has gradually decreased (Figure 8). There were two peaks in WVCs involving Siberian weasels during March–April and September–November (Figure 8). On Jeju Island, Siberian weasels were the most frequently road-killed species, accounting for 35% of total WVCs on the island, while the species represented 6–19% of total WVCs on the mainland of South Korea.

The top 10 models for Siberian weasels consistently included road width, altitude, traffic volume, and month. Urbanization occurred in eight models, and the distance from vegetation was confirmed in six models. The road shoulders appeared in five models, followed by both the median strip and vegetation rank (four models). However, wildlife fences were excluded from all of the top 10 models (Table 5). The presence of median strips, road width, altitude, traffic volume, vegetation rank, and distance from vegetation were negatively related to the number of Siberian weasel WVCs, whereas road shoulders and urbanization were positively related.

### 3.4. WVCs Involving Water Deer

**Figure 9:**
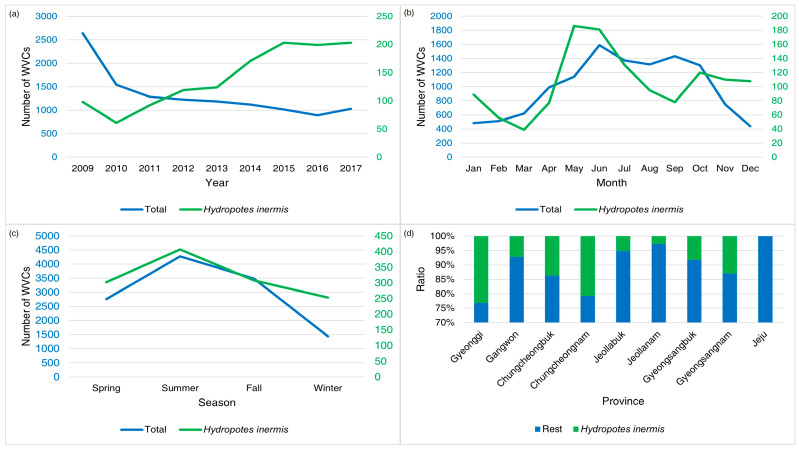
The annual (**a**), monthly (**b**), seasonal (**c**), and provincial (**d**) patterns of wildlife–vehicle collisions (WVCs) involving water deer (*Hydropotes inermis*) from 2009 to 2017 in South Korea. The blue numbers indicate total WVCs and the green numbers indicate WVCs of water deer.

Since the decline in 2010, WVCs involving water deer have continuously increased (Figure 9). In the monthly trend for water deer WVCs, the peaks occurred in May and June (Figure 2 and Figure 9). When considering the seasons, WVCs involving water deer during summer were 1.5 times higher than in winter (Figure 9). Except on Jeju Island, where water deer are absent, WVCs involving water deer accounted for 2–30% of the total number of WVCs. Between latitudes 36 and 37 degrees, more WVCs of water deer were reported.

Except for road shoulders, altitude, and traffic volume, other variables appeared in all of the top four selected models for water deer. Road shoulders and altitude appeared in three out of the five models, and traffic volume was included in only one model. All road variables, except traffic volume, were positively related to the WVCs involving water deer (Table 6).

### 3.5. WVCs Involving Red Squirrels

**Figure 10:**
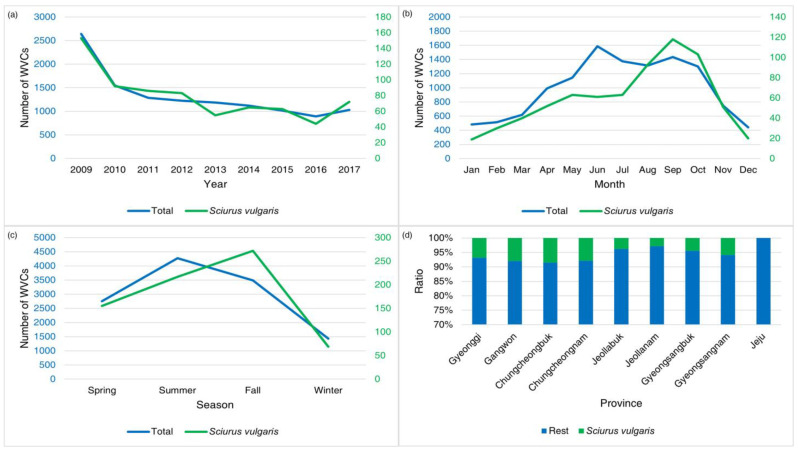
The annual (**a**), monthly (**b**), seasonal (**c**), and provincial (**d**) patterns of wildlife–vehicle collisions (WVCs) involving red squirrels (*Sciurus vulgaris*) from 2009 to 2017 in South Korea. The blue numbers indicate total WVCs and the green numbers indicate WVCs of red squirrels.

WVCs involving red squirrels showed a declining trend, but there were two upswings in 2014 and 2017 (Figure 10). The monthly peaks for WVCs involving red squirrels were during August and October (Figure 10). A pattern of red squirrel WVCs consistently increased from spring through autumn, followed by a substantial decline during winter (Figure 3 and Figure 10).

The top 10 models for red squirrels consistently included road shoulder, altitude, distance from vegetation, and month. Speed limit appeared in nine models, while road width was included in three models, followed by both traffic volume and vegetation rank (two models). The presence of median strips, wildlife fences, and urbanization were each included in only one model (Table 7).

### 3.6. WVCs Involving Korean Hares

**Figure 11:**
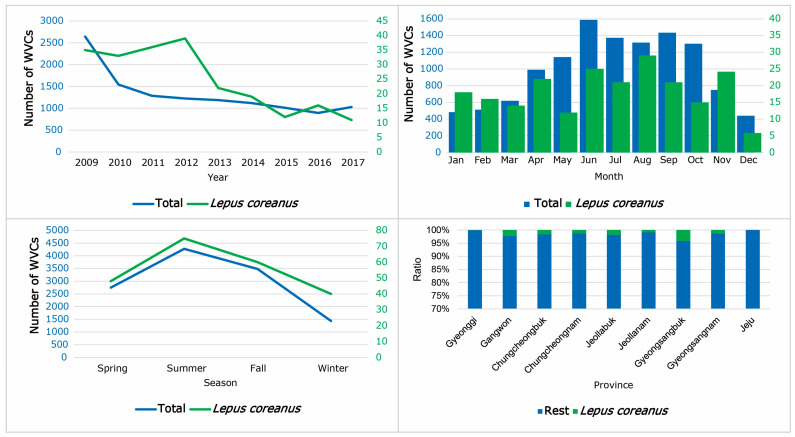
The annual, monthly, seasonal, and provincial patterns of wildlife–vehicle collisions (WVCs) for Korean hare (*Lepus coreanus*) from 2009 to 2017 in South Korea. The blue numbers indicate total WVCs and the green numbers indicate WVCs of Korean hares.

Until 2012, there was an increase in Korean hare WVCs, but since 2013, the numbers of hare WVCs have consistently declined (Figure 11). The highest number of WVCs involving Korean hares occurred in August, while December had the lowest count, although a distinct monthly pattern for WVCs involving Korean hares was not clear (Figure 11).

For Korean hare WVCs, the top 11 models consistently included the distance from vegetation, altitude, and month. Median strip was included in 10 models, followed by speed limit in five models. Both road width and urbanization were included in three models, and road shoulders and wildlife fences each appeared in only one model, while vegetation rank was excluded from all selected models (Table 8). While the distance from vegetation, road width, and wildlife fences were negatively related, other variables were positively related to hare road-kills (Table 8).

### 3.7. WVCs Involving Leopard Cats

**Figure 12:**
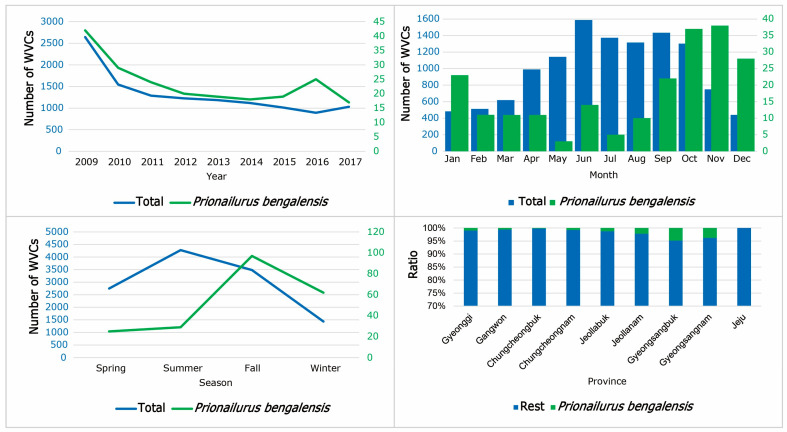
The annual, monthly, seasonal, and provincial pattern of WVCs (wildlife–vehicle collisions) for Leopard cat (*Prionailurus bengalensis*) from 2009 to 2017 in South Korea. The blue numbers indicate total WVCs and the green numbers indicate WVCs of leopard cats.

Although there was an increase from 2014 to 2016, WVCs involving leopard cats showed a declining pattern (Figure 12). The peak in WVCs involving leopard cats was observed in November, with the lowest numbers in May (Figure 12). Seasonally, WVCs involving leopard cats were most prevalent during autumn. In contrast to the six other species, leopard cats had a unique seasonal pattern, with more WVCs during winter compared to spring and summer (Figure 12). At latitude 36 degrees, WVCs involving leopard cats were at least 1.5 times higher than at other latitudes.

Among the top 10 models for leopard cat WVCs, traffic volume, vegetation rank, and month were included in all models, followed by both speed limit and altitude (nine models), road width (five models), and wildlife fence (four models). The distance from vegetation, road shoulder, and urbanization were excluded from all 10 models (Table 9). While altitude, traffic volume, road width, and vegetation rank were negatively related, the presence of median strips, wildlife fences, and speed limit were positively related to WVCs involving leopard cats (Table 9).

## 4. Discussion

In South Korea, nearly half of all WVCs from 2009 to 2017 were concentrated in four species: raccoon dogs (average national density in South Korea from 2009 to 2017 was 3.77 individuals/ km^2^ ± standard error 0.44 by National Wildlife Survey), Siberian chipmunks (6.46 individuals/ km^2^ ± 0.80), Korean hares (3.33 individuals/km^2^ ± 1.33), and water deer (7.44 individuals/ km^2^ ± 0.66). The higher frequency of WVCs in these four species might be due to their higher population densities. While the WVCs involving raccoon dogs and Korean hares were more widely distributed across the country, WVCs involving Siberian chipmunks and water deer were concentrated regionally. The decreasing trend in total WVCs in South Korea appeared to be primarily a result of prevention measures rather than fluctuations in wildlife population densities, based on national wildlife surveys from the same period [34]. The WVC prevention efforts included installing wildlife fences, constructing corridors, installing strategically placed road signs in wildlife crossing areas, conducting extensive awareness campaigns for drivers, and guidance through vehicle GPS navigation [35]. Since the government initiated a recovery plan for ecological networks in 2010 [36], wildlife corridors and other infrastructure for preventing WVCs have been established across the country [35].

The seasonality of WVCs can be explained by ecological and behavioral features of species, such as increased animal movement during breeding and dispersal seasons and winter torpor or hibernation [10,37,38,39,40,41]. Spatial differences in WVCs seem to be more closely related to available wildlife habitats rather than the expansion of road networks or increased traffic [37,42].

Based on linear mixed-effects models for total WVCs in South Korea, six variables were consistently included in the top six selected models (Table 2). Wildlife fences were negatively associated with the number of WVCs, suggesting their effectiveness in collision prevention. However, for raccoon dogs, the most common road-killed species, wildlife fences were positively correlated with WVCs, possibly due to their installation in high-risk areas [42,43,44] Also, inefficient fences may actually lead to incidents. Although wildlife fences should be constraints that wildlife cannot cross or circumnavigate, they can also act as ‘obstacles’ that prevent wildlife from escaping from roads [45].

The presence of road shoulders might facilitate the approaches of animals to roads. The higher speed limits causing more WVCs is plausible. The number of road lanes displayed a negative correlation with WVCs (Table 2). In other words, narrower roads with fewer lanes correlated with more WVCs. First, narrow roads could lure wildlife into crossing the roads more readily. Second, wider roads with more lanes might have more effective WVC prevention measures, making them less accessible to wildlife. Third, narrow roads could be located more closely to wildlife habitats than wider roads.

The speed limits and median strips included in six models respectively showed positive and negative relationships with WVCs. This suggests that higher speeds can cause more WVCs and median strips (central structures) can prevent wildlife crossing. Roads with shoulders experienced a higher frequency of WVCs since road shoulders let wildlife access roads more readily.

Higher altitudes and less urbanized areas (lower human footprint index) with more disturbed vegetation (higher vegetation rank) were also positively correlated with WVCs. This indicates that more WVCs occurred at higher elevations and in less-developed areas with more-disturbed vegetation. In Korea, wildlife habitats are often located in mountainous and sparsely human-populated areas, and major roads tend to be situated near disturbed rather than well-preserved vegetation areas.

The top seven mammal species involved in South Korean WVCs displayed different WVC patterns and tendencies (Figure 2). Raccoon dogs, with the highest number of WVCs, exhibited a distinctive pattern. The number of raccoon dog road-kills significantly decreased from December and remained lower until May. This seasonality is likely due to the winter torpor (hibernation) of raccoon dogs, which leads to reductions in their overall activity, including venturing onto roadways [12]. Since raccoon dogs, particularly those that have recently given birth, disperse, expanding their home range from late May to mid-June, this dispersion leads to more WVCs [46]. Because raccoon dogs are widely distributed across the Korean Peninsula with high density [12], WVCs involving the animal show small differences spatially across different administrative regions or latitudes. However, in contrast to the general WVC pattern, WVCs involving raccoon dogs were more concentrated on wider roads with higher speed limits and a median strip. The WVC models of raccoon dogs suggest that this species is less deterred from accessing larger roadways with fast traffic [16].

Similarly to raccoon dogs, Siberian chipmunks also displayed a drastic decline in WVCs from November, likely due to winter torpor, followed by an increase during May. As Siberian chipmunks emerge from hibernation between late April and May in Korea, their activity is rapidly increased due to birth and subsequent dispersal. WVCs involving Siberian chipmunks were concentrated in specific regions, particularly along roads in forests. The majority of WVC reports for chipmunks came from KNPS. Wider roads with more lanes and WVC mitigation measures such as wildlife fences and median strips accounted for fewer WVCs involving chipmunks. Another explanation is that those wider roads are simply more distant from chipmunk habitats. On the contrary, narrower roads with slower speed limits and no shoulders, commonly found in forested areas, exhibited higher rates of WVCs for Siberian chipmunks. This result might be explained by their closer proximity to chipmunk habitats [47]. Wildlife fences could not deter this small animal.

The Siberian weasel showed a distinct pattern of WVCs, with a clear increase during spring and autumn. This seasonal pattern of weasel WVCs corresponds to the mating season, which typically occurs in March and April, leading to increased activity [12]. During autumn, young weasels become more independent and disperse, resulting in increased WVCs. Due to the different species composition of mammalian fauna on the island, Siberian weasels were the only species among the top seven WVC mammals on Jeju Island. Except on Jeju Island, the WVCs involving Siberian weasels across the country showed uniform patterns regardless of administrative or latitudinal regions. Unlike models of other species, the road shoulders were positively related to WVCs involving Siberian weasels. Road shoulders might allow Siberian weasels easier access when approaching roads.

While total WVCs had a continuous declining pattern, water deer displayed an increasing pattern of WVCs until 2017. The population density of water deer per 10 hectares increased from 6.6 in 2009 to 8.3 in 2017, indicating a strong relationship between WVCs and density [27,34]. WVCs involving water deer were most common in May and October, which are respectively dispersal and mating seasons [16,48]. WVCs involving water deer were concentrated in Gyeonggi and Chungcheongnam Provinces. The concentration of water deer WVCs in these two provinces could be linked to the habitat preference of water deer for low wetlands [12]. Contrary to the general trends, WVCs involving water deer were more frequent on wider roads with median strips, wildlife fences, and road shoulders. The models of water deer WVCs suggest that (1) these deer do not avoid wider roads, (2) fences are efficient in preventing water deer from jumping into the roads, (3) wildlife fences and central fences might trap the animal on the road, and (4) road shoulders might provide easy access to roads [49].

Red squirrels experienced a peak in WVCs in September, likely due to their foraging habits. During September and October, these squirrels become highly active in collecting their food resources, such as conifer cones and walnuts [12]. The red squirrel WVCs during these months should be attributed to this increased foraging during harvesting season. While WVCs involving red squirrels accounted for less than 10% of the total WVCs in South Korea, in Gangwon Province, there were 2–7 times more WVCs involving red squirrels than in other provinces. This regional difference can be explained by the red squirrel’s arboreal inhabitance [50,51]. Despite the small proportion of overall WVCs, the linear mixed-effects models of red squirrel WVCs displayed trends similar to the total WVC pattern, but the presence of road shoulders was negatively related. This arboreal squirrel might avoid more open roads having road shoulders.

From 2009 to 2011, the WVCs involving Korean hares showed an increasing trend, but between 2012 and 2017, the number of hare WVCs sharply declined to about one-third. This drastic change in WVCs involving Korean hares might have been caused by a significant decline in the Korean hare population [52]. The WVCs involving Korean hares did not show clear seasonality, likely due to the irregular mating and birthing seasons of Korean hares [12]. Unlike WVCs involving other animals, Korean hare WVCs were positively related to median strips, wildlife fences, and road shoulders. For Korean hares, these road structures might hinder their escape from oncoming traffic.

The WVCs involving leopard cats exhibited a peak in September, which overlapped with this species’ mating season [12]. Unlike the patterns of total WVC models, the models for leopard cats showed positive correlations with median strips and wildlife fences. These mitigation structures may not effectively prevent the crossing of leopard cats. Since leopard cats often move along drainage pipes and culverts under road fences or cross roads directly through gaps in fences, the efficacy of median strips and wildlife fences as prevention measures for this species is limited [53,54]. Rather, these road structures may confine the cats on the roads, once they have entered. This small cat might avoid wider roads but could be readily killed on high-speed roadways.

Overall, the WVC models differed among species (Table 10). Median strips generally have a negative impact, acting as ecological traps for animals on roads. Wildlife fences can reduce WVCs for some species but may hinder road escape for others, such as raccoon dogs and Korean hares. Road shoulders may facilitate road access, while speed limits, road width, and traffic volume contribute to more WVCs but can deter small species like Siberian weasels from approaching roads. Reducing vehicle speeds may be effective for larger animals [4,55]. Therefore, the effectiveness of wildlife fences on WVCs may increase depending on the characteristics of the target species [56,57,58]. Peripheral vegetation impacts WVCs differently by species. While well-preserved vegetation (lower vegetation rank) correlated with more WVCs for Siberian weasels, water deer, red squirrels, and leopard cats, more disturbed vegetation (higher vegetation rank) was associated with more WVCs for smaller species such as the Siberian chipmunk and Korean hare. While raccoon dogs, Siberian chipmunks, Siberian weasels, red squirrels, and Korean hares experienced WVCs relatively close to vegetation, only water deer WVCs tended to occur more frequently at greater distances from vegetation.

The South Korean government has attempted to mitigate WVCs through the construction of ecological corridors and wildlife fences [35]. In 1998, ecological corridors were first introduced in Jirisan National Park, South Korea. Despite active construction of corridors since then, their effectiveness in WVC prevention has been rarely measured, and essential ecological data and technical resources for WVC management have been limited [36,59,60,61,62]. Although some wildlife corridors were initially constructed inappropriately [63], certain wildlife corridors have efficiently mitigated WVCs [64]. The active movements of Korean hare, raccoon dog, and water deer on some artificial corridors with dense vegetation have been identified [61]. After the installation of wildlife fences, WVCs were reduced by an average of 43.5% along a 45 km section of the Joongang Highway [65]. Since 2010, active campaigns for reducing WVCs have been implemented by the government, and guidelines for WVC prevention structures have been established [63,64]. However, WVCs in South Korea have occurred continuously and remain a serious social issue [35].

Despite the high occurrences of WVCs, small- to medium-sized animals have rarely been considered for WVC mitigation efforts due to the relatively minor damage they cause to vehicles. Unlike those involving other species, water deer WVCs have increased, garnering attention from wildlife scientists and road engineers [65]. Although water deer have been regarded as a focal species for WVCs in South Korea, mitigation efforts for this species have not been effectively implemented [49]. Rather than constructing expensive corridors such as eco-bridges, this research suggests that the mitigation strategy for water deer needs be reevaluated, with particular attention to seasonality and a focus on larger roads for intensified mitigation efforts such as thorough prevention of penetration into the roads.

## 5. Conclusions

Clear seasonal and species-specific differences in WVCs were identified in our study. Road features had varying effects on WVCs among different species. Interestingly, wildlife fences were sometimes positively correlated with WVCs for certain species, while high speeds were negatively correlated for others. Our study indicates that WVCs are not solely associated with the absence/presence of prevention structures like wildlife corridors, fences, or median strips. Instead, the data reveal that species-specific characteristics, seasonality, peripheral vegetation, and road types also play a significant role in WVCs models. There is no universal solution for reducing WVCs across all species. Effective WVC mitigation strategies must consider the interactions between road characteristics and target wildlife species. This approach ensures more effective and customized prevention measures.

## Figures and Tables

**Table 1:** The number of latitudinal wildlife–vehicle collisions (WVCs) for seven major mammal species (>1% of contribution) from 2009 to 2017 in South Korea with the length of survey roads.

Latitude	*Nyctereutes procyonoides*	*Eutamias sibiricus*	*Mustela sibirica*	*Hydropotes inermis*	*Sciurus vulgaris*	*Lepus coreanus*	*Prionailurus bengalensis*	Length of Survey Roads (km)
33°	0	0	120	0	0	0	0	252.71
34°	241	90	278	40	53	16	37	409.99
35°	440	755	247	205	128	48	59	1492.38
36°	494	244	318	500	236	87	89	1726.77
37°	306	395	259	440	234	56	19	1387.56
38°	62	54	52	85	62	16	9	212.83

**Table 2:** Top six linear mixed-effects models of wildlife–vehicle collisions (WVCs) and road variables based on field data collected from 2009 to 2017 in South Korea. Models were selected by cAIC (ΔAIC < 2).

Rank	Median Strip	Wildlife Fence	Road Shoulder	Road Speed	Road Width	Altitude	Traffic Volume	Vegetation Rank	Distance from Vegetation	Urbanization	cAIC	ΔAIC
1	-	−0.169	−0.247	0.003	−0.066	0.042	-	0.221	-	−0.117	51,647.3	0
2	-	−0.170	−0.241	-	−0.047	0.038	-	0.217	-	−0.112	51,647.6	0.28
3	0.050	−0.176	−0.244	-	−0.054	0.039	-	0.217	-	−0.114	51649.1	1.72
4	0.033	−0.173	−0.248	0.003	−0.069	0.042	-	0.221	-	−0.118	51,649.1	1.78
5	-	−0.169	−0.248	0.003	−0.068	0.043	0.003	0.220	-	−0.121	51,649.2	1.82
6	-	−0.169	−0.247	0.003	−0.066	0.042	-	0.209	0.004	−0.118	51,649.2	1.9
Avg.	-	−0.17	−0.25	0.003	−0.066	0.042	-	0.22	-	−0.12	-	-

**Table 3:** Top 11 linear mixed-effects models of wildlife–vehicle collisions (WVCs) involving raccoon dogs (*Nyctereutes procyonoides*) and road variables based on field data collected from 2009 to 2017 in South Korea. Models were selected by cAIC (ΔAIC < 2). ‘+’ means having several values.

Rank	Median Strip	Wildlife Fence	Month	Road Shoulder	Road Speed	Road Width	Altitude	Traffic Volume	Vegetation Rank	Distance from Vegetation	Urbanization	cAIC	ΔAIC
1	-	-	+	-	0.00066	-	−0.0024	-	-	−0.0021	0.007	1349	0
2	-	-	+	-	0.00069	-	−0.0027	−0.0009	-	−0.0021	0.009	1349.5	0.51
3	0.007	-	+	-	0.00060	-	−0.0023	-	-	−0.0022	0.006	1349.8	0.75
4	0.008	-	+	-	0.00062	-	−0.0026	−0.0010	-	−0.0021	0.009	1349.9	0.89
5	-	-	+	-	0.00073	-	−0.0028	-	-	−0.0021	-	1349.9	0.91
6	0.008	-	+	-	0.00065	-	−0.0027	-	-	−0.0021	-	1350.1	1.07
7	-	−0.003	+	-	0.00065	-	−0.0024	-	-	−0.0021	0.007	1350.5	1.53
8	-	-	+	−0.004	0.00066	-	−0.0024	-	-	−0.0021	0.007	1350.6	1.6
9	-	-	+	-	0.00066	-	−0.0024	-	0.002	−0.0026	0.007	1350.7	1.69
10	-	-	+	-	0.00062	0.001	−0.0024	-	-	−0.0022	0.006	1350.8	1.81
11	0.008	−0.004	+	-	0.00058	-	−0.0023	-	-	−0.0022	0.006	1350.9	1.93
Avg.	-	-	-	-	0.00066	-	−0.0024	-	-	−0.0021	0.007	-	-

**Table 4:** Top 13 linear mixed-effects models of wildlife–vehicle collisions (WVCs) involving Siberian chipmunks (Eutamias sibiricus) and road variables based on field data collected from 2009 to 2017 in South Korea. Models were selected by cAIC (ΔAIC < 2). ‘+’ means having several values.

Rank	Median Strip	Wildlife Fence	Month	Road Shoulder	Road Speed	Road Width	Altitude	Traffic Volume	Vegetation Rank	Distance from Vegetation	Urbanization	cAIC	ΔAIC
1	-	0.0070	+	−0.0113	−0.0021	-	0.0114	-	0.008	−0.00168	-	−2315.8	0
2	-	0.0068	+	−0.0114	−0.0022	0.0026	0.0115	-	0.008	−0.00173	-	−2315.7	0.15
3	-	0.0070	+	−0.0113	−0.0021	-	0.0113	-	-	-	-	−2314.9	0.93
4	0.005	0.0063	+	−0.0116	−0.0021	-	0.0115	-	0.008	−0.00170	-	−2314.8	1.04
5	-	0.0069	+	−0.0115	−0.0022	0.0026	0.0115	-	-	-	-	−2314.7	1.09
6	-	-	+	−0.0113	−0.0021	-	0.0115	-	0.008	−0.00169	-	−2314.4	1.41
7	-	-	+	−0.0114	−0.0022	0.0027	0.0116	-	0.008	−0.00174	-	−2314.4	1.42
8	-	0.0071	+	−0.0113	−0.0021	-	0.0113	-	0.002	-	-	−2314.3	1.54
9	0.007	-	+	−0.0117	−0.0022	-	0.0116	-	0.008	−0.00172	-	−2314.1	1.69
10	0.003	0.0064	+	−0.0116	−0.0022	0.0023	0.0116	-	0.008	−0.00173	-	−2314	1.76
11	-	0.0069	+	−0.0115	−0.0022	0.0025	0.0115	-	0.002	-	-	−2313.9	1.9
12	-	0.0069	+	−0.0114	−0.0022	0.0028	0.0115	-	0.008	−0.00172	−0.00167	−2313.9	1.96
13	-	0.0070	+	−0.0113	−0.0021	-	0.0114	0.00007	0.008	−0.00168	-	−2313.8	1.99
Avg.	-	0.007	-	−0.011	−0.002	-	0.011	-	0.008	−0.0017	-	-	-

**Table 5:** Top 10 linear mixed-effects models of wildlife–vehicle collisions (WVCs) involving Siberian weasels (*Mustela sibirica*) and road variables based on field data collected from 2009 to 2017 in South Korea. Models were selected by cAIC (ΔAIC < 1). ‘+’ means having several values.

Rank	Median Strip	Wildlife Fence	Month	Road Shoulder	Road Speed	Road Width	Altitude	Traffic Volume	Vegetation Rank	Distance from Vegetation	Urbanization	cAIC	ΔAIC
1	-	-	+	-	-	−0.0079	−0.0063	−0.0015	-	−0.0016	0.0074	−306.2	0
2	-	-	+	0.0078	-	−0.0080	−0.0063	−0.0016	-	−0.0015	0.0073	−306	0.15
3	−0.0080	-	+	0.0082	-	−0.0070	−0.0063	−0.0015	-	−0.0015	0.0076	−305.9	0.25
4	−0.0076	-	+	-	-	−0.0070	−0.0064	−0.0015	-	−0.0016	0.0077	−305.8	0.31
5	-	-	+	-	-	−0.0080	−0.0063	−0.0015	−0.0061	-	0.0073	−305.8	0.34
6	-	-	+	0.0078	-	−0.0081	−0.0063	−0.0016	−0.0061	-	0.0072	−305.7	0.49
7	−0.0082	-	+	0.0083	-	−0.0071	−0.0064	−0.0015	−0.0061	-	0.0075	−305.6	0.52
8	−0.0077	-	+	-	-	−0.0071	−0.0064	−0.0015	−0.0061	-	0.0076	−305.6	0.59
9	-	-	+	-	-	−0.0071	−0.0065	−0.0011	-	−0.0015	-	−305.2	0.92
10	-	-	+	0.0080	-	−0.0072	−0.0065	−0.0011	-	−0.0015	-	−305.2	0.98
Avg.	-	-	-	-	-	−0.0079	−0.0063	−0.0015	-	−0.0016	0.0074	-	-

**Table 6:** Top four linear mixed-effects models of wildlife–vehicle collisions (WVCs) involving water deer (*Hydropotes inermis*) and road variables based on field data collected from 2009 to 2017 in South Korea. Models were selected by cAIC (ΔAIC < 2). ‘+’ means having several values.

Rank	Median Strip	Wildlife Fence	Month	Road Shoulder	Road Speed	Road Width	Altitude	Traffic Volume	Vegetation Rank	Distance from Vegetation	Urbanization	cAIC	ΔAIC
1	0.021	0.0085	+	0.0096	0.0009	0.0072	0.0014	-	−0.0115	0.0028	0.0088	−1233.6	0
2	0.021	0.0085	+	-	0.0009	0.0072	0.0014	-	−0.0116	0.0029	0.0090	−1232.5	1
3	0.021	0.0085	+	0.0098	0.0009	0.0075	0.0013	−0.0006	−0.0115	0.0028	0.0102	−1232.3	1.22
4	0.020	0.0092	+	0.0095	0.0008	0.0069	-	-	−0.0116	0.0029	0.0069	−1231.8	1.7
Avg.	0.021	0.0085	-	0.0096	0.0009	0.0072	0.0014	-	−0.012	0.0028	0.0088	-	-

**Table 7:** Top 10 linear mixed-effects models of wildlife–vehicle collisions (WVCs) involving red squirrels (*Sciurus vulgaris*) and road variables based on field data collected from 2009 to 2017 in South Korea. Models were selected by cAIC (ΔAIC < 2). ‘+’ means having several values.

Rank	Median Strip	Wildlife Fence	Month	Road Shoulder	Road Speed	Road Width	Altitude	Traffic Volume	Vegetation Rank	Distance from Vegetation	Urbanization	cAIC	ΔAIC
1	-	-	+	−0.0096	0.0002	-	0.0020	-	-	−0.0029	-	−6641.5	0
2	-	-	+	−0.0094	0.0003	−0.002	0.0018	-	-	−0.0028	-	−6641.5	0.05
3	-	-	+	−0.0096	0.0003	−0.003	0.0020	0.0004	-	−0.0028	-	−6640.1	1.47
4	-	-	+	−0.0089	-	-	0.0014	-	-	−0.0029	-	−6640.1	1.49
5	-	-	+	−0.0096	0.0002	-	0.0020	-	−0.002	−0.0025	-	−6639.9	1.65
6	-	-	+	−0.0094	0.0003	−0.002	0.0018	-	−0.002	−0.0024	-	−6639.9	1.69
7	−0.002	-	+	−0.0094	0.0002	-	0.0019	-	-	−0.0029	-	−6639.8	1.79
8	-	-	+	−0.0096	0.0002	-	0.0020	0.0002	-	−0.0029	-	−6639.6	1.9
9	-	-	+	−0.0095	0.0002	-	0.0019	-	-	−0.0029	−0.0010	−6639.6	1.9
10	-	0.0003	+	−0.0096	0.0002	-	0.0020	-	-	−0.0029	-	−6639.6	1.99
Avg	-	-	-	−0.0096	0.0002	-	0.002	-	-	−0.0029	-	-	-

**Table 8:** Top 11 linear mixed-effects models of wildlife–vehicle collisions (WVCs) involving Korean hares (*Lepus coreanus*) and road variables based on field data collected from 2009 to 2017 in South Korea. Models were selected by cAIC (ΔAIC < 2). ‘+’ means having several values.

Rank	Median Strip	Wildlife Fence	Month	Road Shoulder	Road Speed	Road Width	Altitude	Traffic Volume	Vegetation Rank	Distance from Vegetation	Urbanization	cAIC	ΔAIC
1	0.0055	-	+	-	-	-	0.0019	-	-	−0.0013	-	−20,375.1	0
2	0.0045	-	+	-	0.00009	-	0.0021	-	-	−0.0013	-	−20,374.4	0.64
3	0.0049	-	+	-	-	-	0.0021	-	-	−0.0013	0.0019	−20,374.2	0.87
4	0.0054	-	+	-	0.00013	−0.0012	0.0021	-	-	−0.0012	-	−20,373.8	1.24
5	-	-	+	-	0.00013	-	0.0020	-	-	−0.0013	-	−20,373.7	1.42
6	0.0061	-	+	-	-	−0.0005	0.0019	-	-	−0.0013	-	−20,373.4	1.71
7	0.0057	−0.0010	+	-	-	-	0.0020	-	-	−0.0013	-	−20,373.4	1.72
8	0.0052	-	+	-	0.00012	−0.0015	0.0022	-	-	−0.0013	0.0023	−20,373.3	1.81
9	0.0054	-	+	0.001	-	-	0.0019	-	-	−0.0013	-	−20,373.2	1.84
10	0.0041	-	+	-	0.00007	-	0.0022	-	-	−0.0013	0.0016	−20,373.2	1.92
11	0.0053	-	+	-	-	-	0.0020	0.00009	-	−0.0013	-	−20,373.2	1.92
Avg.	0.0055	-	-	-	-	-	0.0019	-	-	−0.0013	-	-	-

**Table 9:** Top 10 linear mixed effects models of WVCs (wildlife–vehicle collisions) of Leopard cat (*Prio-ilurus bengalensis*) and road variables based on field data collected from 2009 to 2017 in South Korea. Models were selected by cAIC (ΔAIC < 1.4). ‘+’ means having several values.

Rank	Median Strip	Wildlife Fence	Month	Road Shoulder	Road Speed	Road Width	Altitude	Traffic Volume	Vegetation Rank	Distance from Vegetation	Urbanization	cAIC	ΔAIC
1	0.005	-	+	-	0.00017	−0.0018	−0.0006	−0.0011	−0.0029	-	-	−21,337.9	0
2	0.004	0.002	+	-	0.00018	−0.0018	−0.0007	−0.0011	−0.0029	-	-	−21,337.6	0.3
3	-	0.003	+	-	0.00020	−0.0014	−0.0007	−0.0010	−0.0029	-	-	−21,337.5	0.4
4	-	0.003	+	-	0.00015	-	−0.0007	−0.0011	−0.0029	-	-	−21,337.5	0.42
5	-	-	+	-	0.00014	-	−0.0006	−0.0011	−0.0029	-	-	−21,337.1	0.85
6	-	-	+	-	0.00019	−0.0014	−0.0007	−0.0010	−0.0029	-	-	−21,336.9	0.96
7	0.005	-	+	-	0.00021	−0.0017	-	−0.0009	−0.0029	-	-	−21,336.8	1.07
8	0.003	-	+	-	0.00011	-	−0.0006	−0.0012	−0.0029	-	-	−21,336.8	1.12
9	0.003	0.003	+	-	0.00012	-	−0.0006	−0.0012	−0.0029	-	-	−21,336.6	1.34
10	0.005	-	+	-	-	-	−0.0008	−0.0011	−0.0031	-	-	−21,336.5	1.36
Avg.	0.005	-	-	-	0.00017	−0.0018	−0.0006	−0.0011	−0.0029	-	-	-	-

**Table 10:** Positive or negative effects of various road variables on wildlife–vehicle collisions (WVCs) involving the top seven mammal species in South Korea, analyzed using linear mixed-effects models. ‘+’ means having several values.

Species	Median Strip	Wildlife Fence	Road Shoulder	Road Speed	Road Width	Altitude	Traffic Volume	Vegetation Rank	Distance from Vegetation	Urbanization
***Nyctereutes procyonoides***	+	-	-	+	+	-	-	+	-	+
***Eutamias sibiricus***	+	+	-	-	+	+	+	+	-	-
***Mustela*** ***sibirica***	-	None	+	None	-	-	-	-	-	+
***Hydropotes inermis***	+	+	+	+	+	+	-	-	+	+
***Sciurus*** ***vulgaris***	-	+	-	+	-	+	+	-	-	-
***Lepus*** ***coreanus***	+	-	+	+	-	+	+	None	-	+
***Prionailurus bengalensis***	+	+	None	+	-	-	-	-	None	None
**Total**	+	-	-	+	-	+	+	+	+	-

## Data Availability

Data can be used from annual report of wildlife survey from 2009 to 2017.

## Supplementary Materials

The following supporting information can be downloaded at: https://www.mdpi.com/article/10.3390/ani14203012/s1, Figure S1: Multiple factor analysis of wildlife–vehicle collisions (WVCs) involving seven major mammal species from 2009 to 2017 in South Korea; Table S1: The species list and annual number of wildlife–vehicle collisions (WVCs) from 2009 to 2017 in South Korea.
