# Supplementary figures and images for: Wildlife–Vehicle Collisions and Mitigation: Current Status and Factor Analysis in South Korea
Source: Animals (Basel). 2024 Oct 18;14(20):3012. doi: 10.3390/ani14203012 (PMC11505253; doi:10.3390/ani14203012)

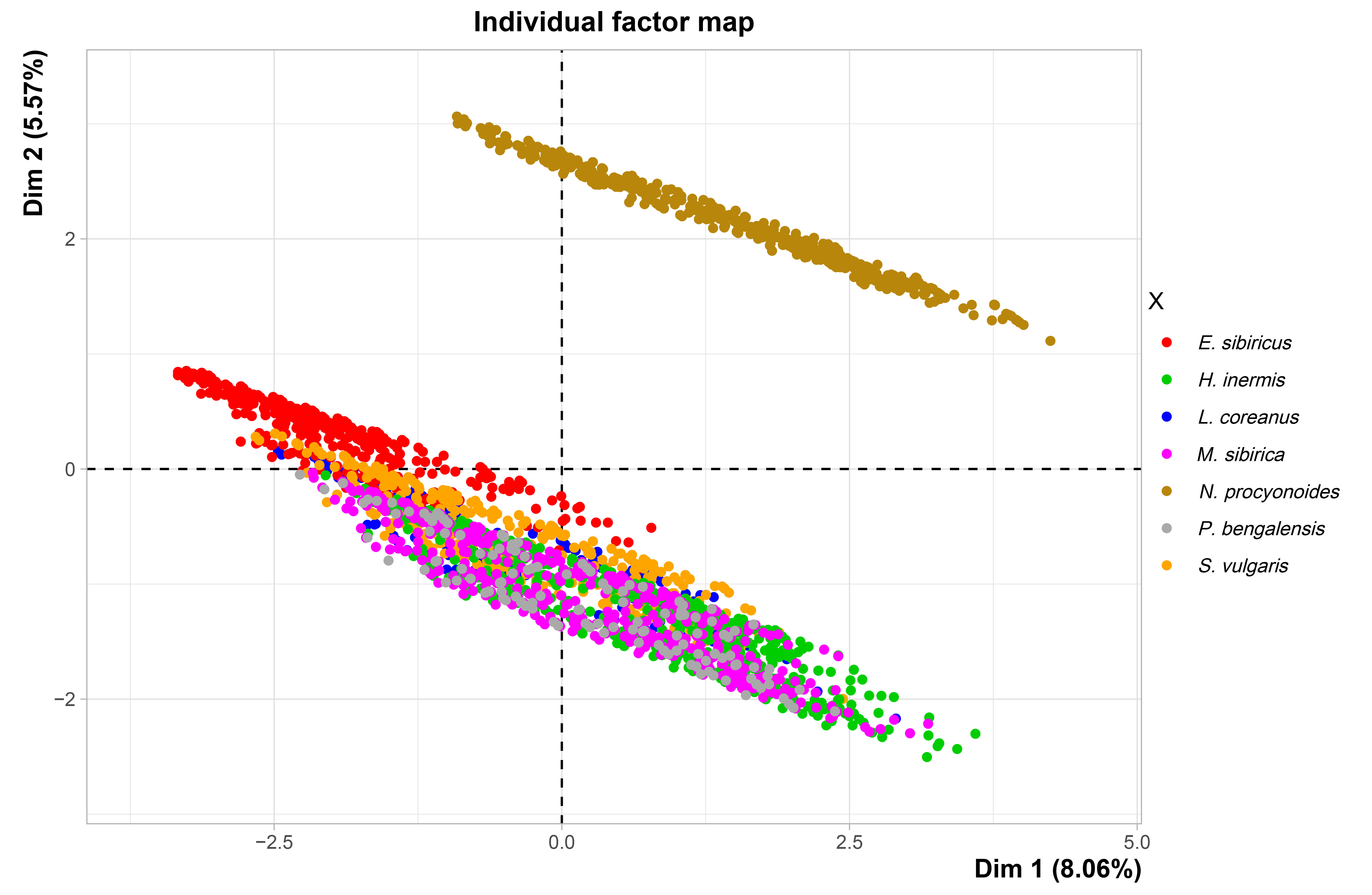

Supplement: Supplementary file 1 [file animals-14-03012-s001.zip › Figure S1.JPEG]
